# Abnormal Expression of Synaptic and Extrasynaptic GABA_A_ Receptor Subunits in the Dystrophin-Deficient *mdx* Mouse

**DOI:** 10.3390/ijms232012617

**Published:** 2022-10-20

**Authors:** Faouzi Zarrouki, Sébastien Goutal, Ophélie Vacca, Luis Garcia, Nicolas Tournier, Aurélie Goyenvalle, Cyrille Vaillend

**Affiliations:** 1 Université Paris-Saclay, CNRS, Institut des Neurosciences Paris Saclay, 91400 Saclay, France; 2 Université Paris-Saclay, UVSQ, Inserm, END-ICAP, 78000 Versailles, France; 3 Université Paris-Saclay, INSERM, CNRS, CEA, Laboratoire d’Imagerie Biomédicale Multimodale (BioMaps), Service Hospitalier Frédéric Joliot, 91401 Orsay, France

**Keywords:** Duchenne muscular dystrophy, GABAergic synapses, PET brain imaging, immunofluorescence, western blots, GABA_A_-receptor clustering, outcome measures

## Abstract

Duchenne muscular dystrophy (DMD) is a neurodevelopmental disorder primarily caused by the loss of the full-length Dp427 dystrophin in both muscle and brain. The basis of the central comorbidities in DMD is unclear. Brain dystrophin plays a role in the clustering of central gamma-aminobutyric acid A receptors (GABA_A_Rs), and its loss in the *mdx* mouse alters the clustering of some synaptic subunits in central inhibitory synapses. However, the diversity of GABAergic alterations in this model is still fragmentary. In this study, the analysis of in vivo PET imaging of a benzodiazepine-binding site radioligand revealed that the global density of central GABA_A_Rs is unaffected in *mdx* compared with WT mice. In contrast, semi-quantitative immunoblots and immunofluorescence confocal imaging in tissue sections revealed complex and differential patterns of alterations of the expression levels and/or clustered distribution of a variety of synaptic and extrasynaptic GABA_A_R subunits in the hippocampus, cerebellum, cortex, and spinal cord. Hence, dystrophin loss not only affects the stabilization of synaptic GABA_A_Rs but also influences the subunit composition of GABA_A_Rs subtypes at both synaptic and extrasynaptic sites. This study provides new molecular outcome measures and new routes to evaluate the impact of treatments aimed at compensating alterations of the nervous system in DMD.

## 1. Introduction

Duchenne muscular dystrophy (DMD) is a lethal X-linked inherited neuromuscular disorder caused by mutations in the gene encoding dystrophin, a cytoskeletal protein normally expressed in both muscles and in the central nervous system (CNS). The DMD syndrome is characterized by progressive muscular degeneration and a neurodevelopmental disorder including a range of central comorbidities, the presence and severity of which depend on the position of the mutations that may alter the expression of several brain dystrophin isoforms encoded by internal promoters [1,2]. The absence of the 427-kDa full-length dystrophin, Dp427, is shared by all patients and is associated with mild cognitive deficits and enhanced emotional reactivity [1,3]. The main mechanistic hypothesis arose from the finding that Dp427 is expressed in principal neurons of brain structures involved in cognitive and behavioral processes, where it colocalizes with a subset of gamma-aminobutyric acid A (GABA_A_) receptors (GABA_A_Rs) [4] and appears to regulate their clustering and/or molecular composition in the postsynaptic densities (PSDs) of central inhibitory synapses [5,6]. These processes are essential for the proper development and function of GABAergic synapses, and their alteration likely plays a role in the etiology of neurodevelopmental disorders by influencing brain connectivity, activity, and plasticity [7].

GABA_A_Rs are GABA-gated, chloride-selective channels located either at the inhibitory synapse mediating fast, phasic synaptic inhibition, or at extrasynaptic sites mediating tonic inhibition [8]. GABA_A_Rs are heteropentameric assemblies that contain at least three different subunits assembled from more than 20 subunits including α1–6, β1–4, γ1–4, δ, ρ1–3, ε, θ, and π. The majority contain pairs of a single type of α- and β-subunit variants, associated with the γ2-subunit required for the binding site of the positive modulator, benzodiazepine. However, minor populations of non-stoichiometric, hybrid receptors containing two different α subunits have also been reported [9,10,11]. Combinations containing α1-3, β, and γ subunits are mostly represented at the synaptic site, while those containing α4-6, β, and δ subunits are mostly extrasynaptic [12,13]. The subunit identity of GABA_A_Rs, which determines their pharmacological and physiological properties, not only depends on their subcellular localization but also varies during development and across brain structures [14,15]. Moreover, various anchoring and trafficking mechanisms regulate the density and size of postsynaptic GABA_A_Rs clusters during synapse formation and maturation, likely in an activity-dependent manner [5,16,17].

Dp427 dystrophin has several molecular partners at the postsynaptic membrane of inhibitory synapses, which together form the dystrophin-associated complex [2] showing interactions with PSD proteins involved in the regulation of GABA_A_Rs clustering, such as the synaptic scaffolding molecule (S-SCAM) and the transsynaptic neurexin-neuroligin complex [18]. However, the impact of Dp427 loss on the density and molecular heterogeneity of GABA_A_Rs is still ill-defined. While Dp427 is dispensable for the initial gephyrin-dependent anchoring of GABA_A_Rs [4], its absence in *mdx* mice, a mouse model of DMD lacking Dp427, is associated with significant decreases in the density and size of clusters containing the α1 subunit in the cerebellum and the α2 subunit in the hippocampus and amygdala [6,19,20]. A series of studies also demonstrated changes in GABAergic inhibitory transmission and their impact on synaptic plasticity and emotional behaviors in this mouse model [19,21,22,23,24,25,26,27,28]. In all, it is believed that the Dp427-associated complex stabilizes large GABA_A_R clusters at the synapse, perhaps by limiting their lateral diffusion, thereby playing a critical role in the molecular and functional long-term maintenance of inhibitory synapses. Interestingly, an electrophysiological study in cerebellar slices [29] and an in vivo pharmacological experiment [23] both revealed an enhanced sensitivity of *mdx* mice to a selective activator of extrasynaptic GABA_A_Rs, suggesting a change in the density and/or composition of these receptors. However, the molecular studies in *mdx* mice have to date been restricted to the α1 and α2 subunits. This should be extended to the expression and distribution of several synaptic and extrasynaptic subunits in various brain structures to better understand the impact of Dp427 loss on inhibitory networks and to identify robust and specific molecular outcome measures for preclinical rescue experiments [20,30,31].

In the present study, we first used in vivo positron emission tomography (PET) imaging of brains from WT and Dp427-deficient (*mdx*) mice with the benzodiazepine-binding site radioligand [^11^C]flumazenil, referred to as a translational tool to investigate disease-induced changes in the GABA_A_R density in both clinical [32,33] and preclinical studies [34,35]. In distinct cohorts of mice, we then undertook a detailed evaluation of expression levels and clustering of a variety of GABA_A_R subunits ex vivo, using semi-quantitative Western blotting (WB) and immunofluorescence (IF) techniques, respectively. We selected brain areas with expected relevance to the *mdx* mouse behavioral phenotype and high expression of Dp427 (hippocampus, cerebellum, and cortex). We also analyzed tissue samples from the cervical spinal cord, as the expression of dystrophins in this part of the nervous system has been largely overlooked despite a demonstrated expression of GABA_A_R subunits [15,36,37], and as putative changes in peripheral inhibition might affect mouse behavior. Our PET imaging study shows that the density of main synaptic GABA_A_Rs is unaffected by dystrophin loss, and therefore insufficient to identify relevant translational readouts. In contrast, fine semi-quantitative analyses of tissue samples and sections reveal a complex pattern of changes in expression and distribution of several synaptic and extrasynaptic GABA_A_R subunits in *mdx* mice.

## 2. Results

### 2.1. In Vivo [^11^C]Flumazenil Brain PET Imaging

**Figure 1:**
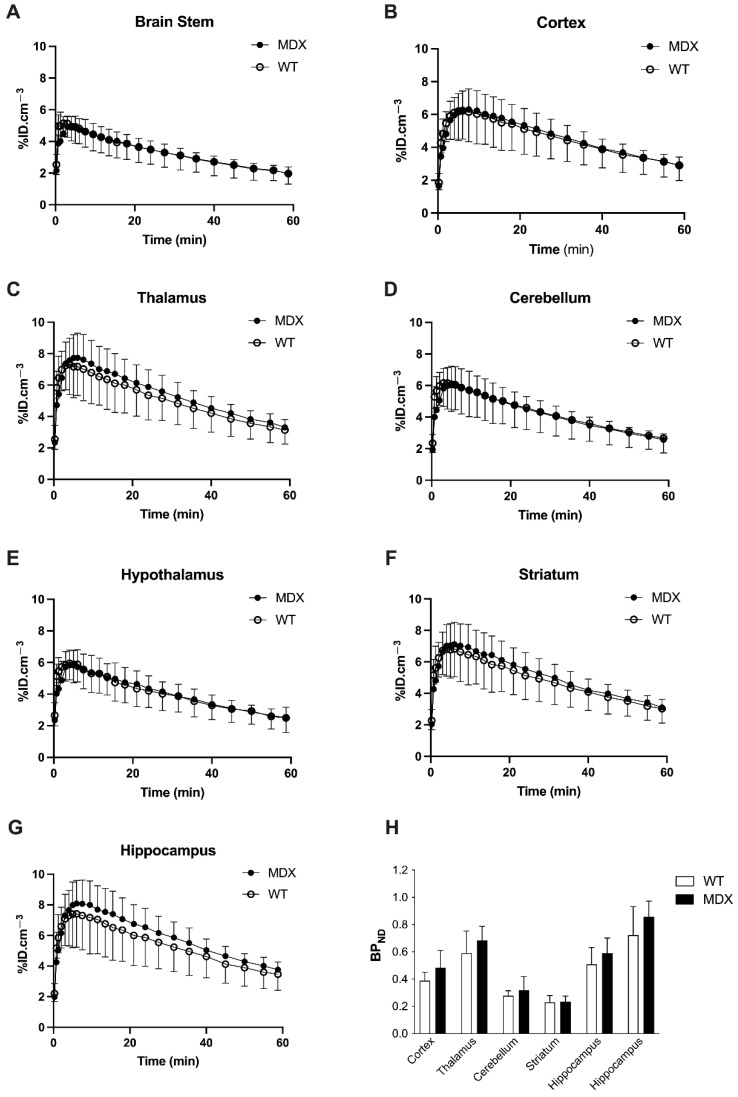
In vivo [11C]flumazenil positron emission tomography (PET) brain imaging. (**A**–**G**) Time-activity curves of [^11^C]flumazenil in the brain stem, cortex, thalamus, cerebellum, hypothalamus, striatum, and hippocampus of wild-type (WT, white symbols and bars, *n* = 5) and *mdx* mice (black symbols and bars, *n* = 6). (**H**) Corresponding binding potential (BP_ND_) to gamma-aminobutyric acid A receptors (GABA_A_Rs), estimated using the brain stem as a reference region. Data are shown as mean ± S.D. Difference in BP_ND_ between WT and *mdx* mice was not significant (All *p* > 0.05).

In both WT and *mdx* mice, higher uptake of [^11^C]flumazenil was observed in GABA_A_R rich regions such as the hippocampus, and lower uptake was seen in the brain stem and hypothalamus (Figure 1). This is consistent with the reported brain distribution of this radioligand in mice [34]. The brain kinetics of [^11^C]flumazenil were strikingly similar in WT and *mdx* mice, in all tested brain regions (Figure 1A–G). There was no significant difference in the area under the time-activity curve of [^11^C]flumazenil between WT and *mdx* mice, in any brain regions, including the brain stem, which was used as a reference to estimate [^11^C]flumazenil binding to GABA_A_Rs (*p* < 0.05). There was no significant genotype difference in the corresponding [^11^C]flumazenil binding potential (BP_ND_) in any tested brain region (all *p* > 0.05; Figure 1H). Voxel-to-voxel analysis was then performed to investigate sub-regional change in [^11^C]flumazenil BP_ND_ and we found no cluster with a significant difference in BP_ND_ between parametric images obtained in WT and *mdx* mice. An unconventional normalization of the BP_ND_ to the striatum was also attempted, as the striatum can be considered as a pseudo-reference structure devoid of dystrophin [6,38], which led to the same conclusion (Supplementary Figure S1). Altogether, no difference in [^11^C]flumazenil binding could be observed in *mdx* compared with control mice, suggesting the absence of macroscopically detectable change in GABA_A_Rs expression.

### 2.2. Western Blot Analyses

**Figure 2:**
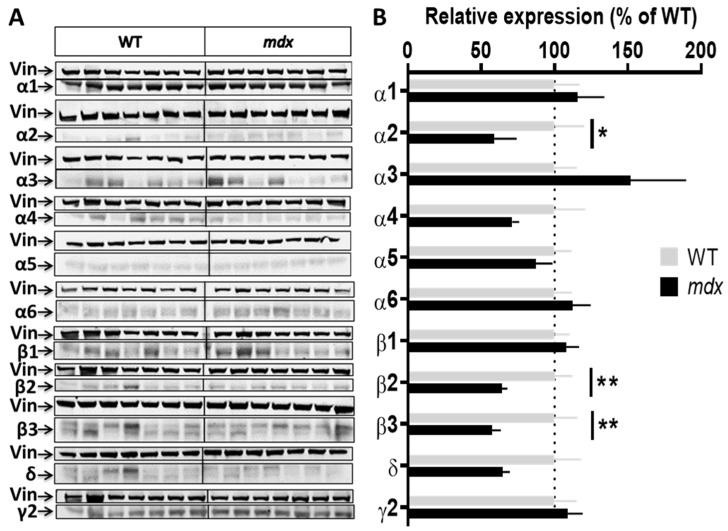
Expression level of GABA_A_R subunits in the hippocampus. (**A**) Immunoblots showing the detection of 11 GABA_A_R subunits in hippocampal extracts from 7 WT and 7 *mdx* mice loaded on the same gels. As indicated, vinculin was used as a loading control for each blot. (**B**) Quantification of each GABA_A_R subunit normalized to WT level (gray bars, dotted line) to reflect their relative expression (% of WT) in *mdx* mice (black bars). Data are shown as mean ± S.E.M.; significant differences between genotypes are shown by asterisks (* *p* < 0.05, ** *p* < 0.01).

**Figure 3:**
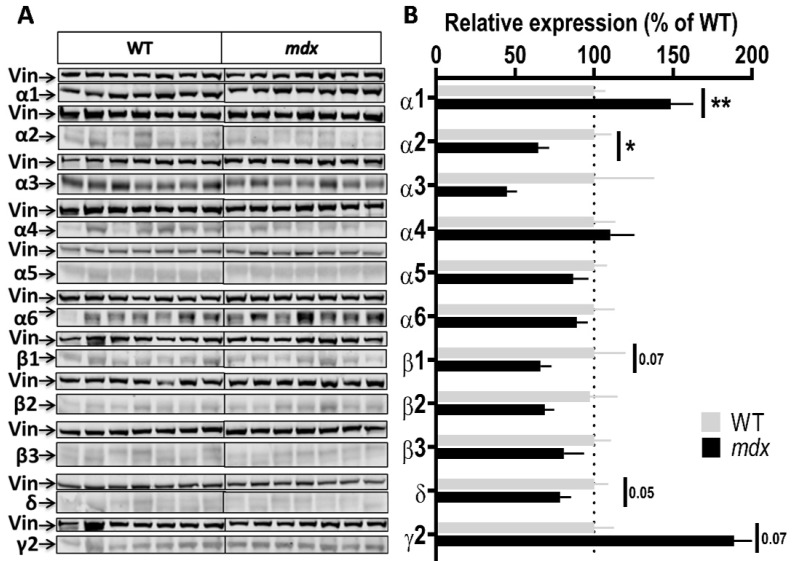
Expression level of GABA_A_R subunits in the cortex. (**A**) Immunoblots showing the detection of 11 GABA_A_R subunits in cortex extracts from 7 WT and 7 *mdx* mice loaded on the same gels. As indicated, vinculin was used as a loading control for each blot. (**B**) Quantification of each GABA_A_R subunit normalized to WT level (gray bars, dotted line) to reflect their relative expression (% of WT) in *mdx* mice (black bars). Data are shown as mean ± S.E.M.; significant differences between genotypes are shown by asterisks (* *p* < 0.05, ** *p* < 0.01). Marginal differences are also indicated (*p* = 0.05 or 0.07).

**Figure 4:**
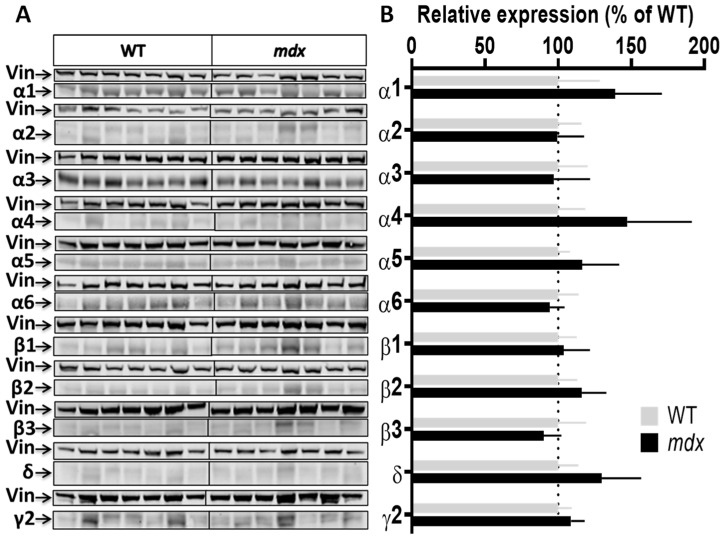
Expression level of GABA_A_R subunits in the cerebellum. (**A**) Immunoblots showing the detection of 11 GABA_A_R subunits in cerebellar extracts from 7 WT and 7 *mdx* mice loaded on the same gels. As indicated, vinculin was used as a loading control for each blot. (**B**) Quantification of each GABA_A_R subunit normalized to WT level (gray bars, dotted line) to reflect their relative expression (% of WT) in *mdx* mice (black bars). Data are shown as mean ± S.E.M.; no significant difference was found between genotypes in this structure.

**Figure 5:**
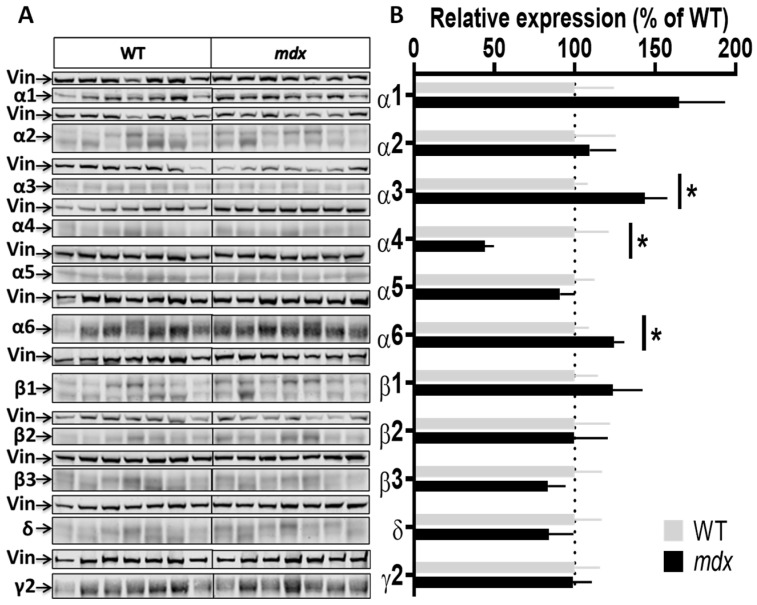
Expression level of GABA_A_R subunits in the cervical spinal cord. (**A**) Immunoblots showing the detection of 11 GABA_A_R subunits in cervical spinal cord extracts from 7 WT and 7 *mdx* mice loaded on the same gels. As indicated, vinculin was used as a loading control for each blot. (**B**) Quantification of each GABA_A_R subunit normalized to WT level (gray bars, dotted line) to reflect their relative expression (% of WT) in *mdx* mice (black bars). Data are shown as mean ± S.E.M.; significant differences between genotypes are shown by asterisks (* *p* < 0.05).

To characterize genotype differences in the expression level of the main subunits composing GABA_A_Rs, we used a panel of antibodies that covers the majority of subunits reported to show large expression in the nervous system (α1, α2, α3, α4, α5, α6, β1, β2, β3, γ2, and δ). We performed a series of semi-quantitative Western blot analyses using protein extracts from the hippocampus, cortex, cerebellum, and spinal cord of the same individuals. To improve the reliability of our comparative quantifications, all samples from a given structure from both genotypes (*n* = 7–8 mice per genotype) were deposited on the same gel and expression levels were then normalized to vinculin. Figure 2, Figure 3, Figure 4 and Figure 5 show the expression levels of GABA_A_R subunits in *mdx* mice expressed as percent of WT mean levels.

Hippocampal samples (Figure 2A,B) from *mdx* mice showed a 40% decrease in the expression level of the α2 subunit (*p* < 0.0205), a 35% decrease for the β2 subunit (*p* < 0.0082), and a 42.5% decrease for the β3 subunit (*p* = 0.0041) as compared with WT, while all other subunits had an expression level comparable to that of WT mice.

Cortex samples (Figure 3A,B) showed a 48.5% increase in the expression level of the α1 subunit (*p* = 0.0037) and, in the opposite, a 35% decrease in the expression level of the α2 subunit (*p* = 0.0175) in *mdx* mice. A trend for a decreased expression was observed for the β1 (*p* = 0.07) and δ (*p* = 0.05) subunits, and for an increased expression for the γ2 subunit (*p* = 0.07), but these genotype differences did not reach significance. In the cerebellum (Figure 4A,B), there was no significant genotype difference in the expression levels of all subunits.

GABA_A_Rs are also detected in the spinal cord [15,36,37] and prior preliminary results suggested that the Dp427 dystrophin could be expressed at low levels in this tissue (not shown). Using the sensitive Jess Western blot system, we demonstrated that Dp427 is expressed in the cervical spinal cord of WT mice, but not in *mdx* mice, and we further determined that its level of expression is 3–4 times lower in this tissue as compared to the cortex (Supplementary Figure S2). Interestingly, we also found significant variations in the expression level of several GABA_A_R subunits in the cervical spinal cord of *mdx* mice (Figure 5A,B), characterized by increases in expression levels for the α3 (40%; *p* = 0.0289) and α6 (25%; *p* = 0.0289) subunits, and conversely, a 56% decrease for the α4 subunit (*p* = 0.0175).

A synthesis of the genotype differences revealed by our Western blot study is shown in Table 1, highlighting the selective structure-dependent alterations in expression levels of GABA_A_R subunits in *mdx* mice.

### 2.3. Immunofluorescence Analyses

A complementary IF study was then undertaken in brain tissue sections (*n* = 5 mice per genotype) to test specific questions that were raised by the Western blot analyses but could not be addressed by this technique.

**Figure 6:**
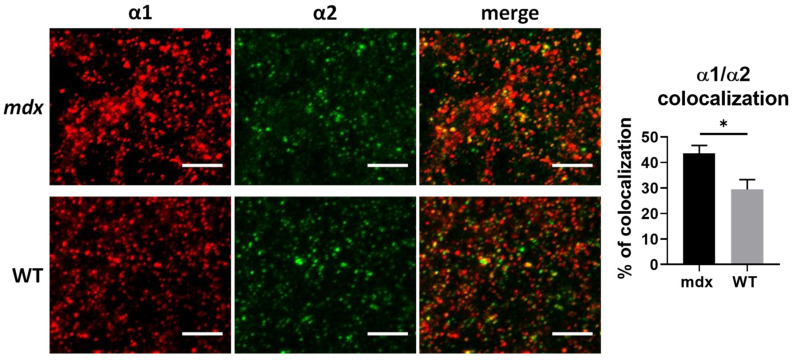
Colocalization of α1 and α2 GABA_A_R subunits in the cortex. The left panel shows representative sample confocal laser scanning microscope images of α1-subunit (red) and α2-subunit (green) immunoreactive signals, as well as merged images of overlaps (yellow dots) between α1-subunit and α2-subunit immunoreactivity in WT and *mdx* mice. The histogram on the right shows the fraction (%) of clusters showing overlaps and reflecting presumed colocalization of these two synaptic subunits. Scale bar: 5 µm. * *p* < 0.05.

First, we wondered whether the opposing changes in the expression levels of α1 and α2 subunits in the cortex of *mdx* mice in the WB study could reflect changes in the proportion of receptors containing both the α1 and α2 subunits, a hypothesis previously suggested by others [18]. To address this question, we analyzed the 3D colocalization of these two subunits in confocal stack images from sensory-motor cortex sections. As shown in Figure 6, more than 20% of α1 and α2 clusters were colocalized in both genotypes, thus suggesting that heteropentamers containing both subunits were indeed present in this region. Interestingly, the percentage of colocalization of α1 and α2 subunits was significantly larger in *mdx* mice (43.57%) than in WT mice (29.42%) (*p* = 0.0317), suggesting a marked change in the subunit composition of GABA_A_Rs in cortical synapses of *mdx* mice.

Our Western blot analyses also suggested that subunits known to be predominantly extrasynaptic (α4, α5, α6, and δ) were expressed at comparable levels in brain structures of the two genotypes, while α4 and α6 subunit expression was significantly and selectively altered in the spinal cord. This was inconsistent with a previous report showing that cerebellar Purkinje neurons of *mdx* mice have an enhanced sensitivity to a selective pharmacological activator of extrasynaptic GABA_A_Rs [29]. We therefore hypothesized that fine alterations in the distribution of these receptors in brain structures could not be detected by a global protein quantification from tissue extracts. To test this hypothesis, we investigated the number, size, and distribution of clusters of α4, α5, and α6 subunits in sections of different brain structures using IF.

**Figure 7:**
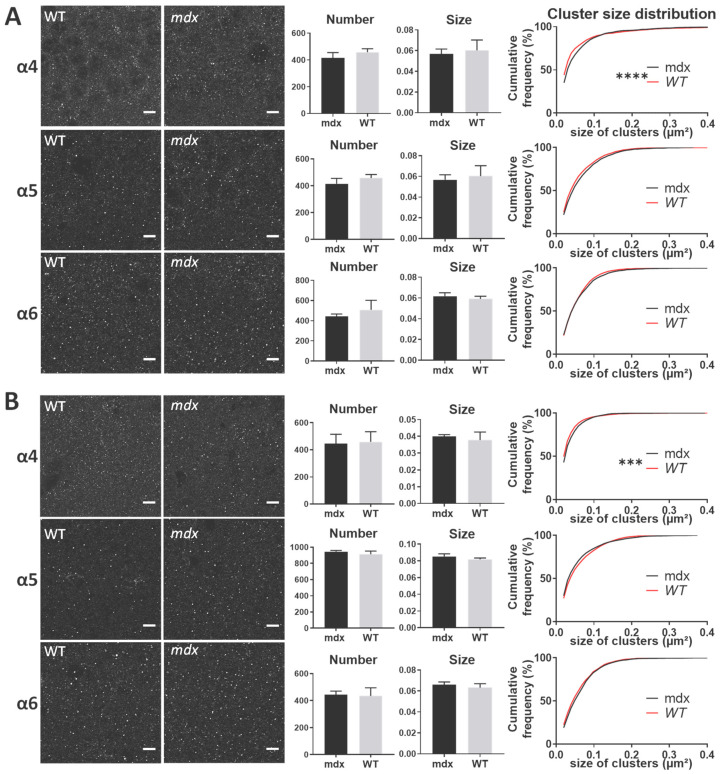
Immunohistological analysis of the α4–6 subunits contained in extrasynaptic GABA_A_Rs in the hippocampus. (**A**) Analysis in the SP. (**B**) Analysis in the SR. For each subunit and for both genotypes, from left to right: Representative sample confocal laser scanning microscope images, mean number of clusters, mean cluster size, and distribution curve of the cluster sizes expressed as cumulative frequency (%). Scale bar: 7 µm. **** *p* < 0.0001, *** *p* < 0.001.

In all structures, the α4, α5, and α6 subunits showed a punctiform immunoreactivity, typical of GABA_A_R cluster labeling. In both the *stratum pyramidale* (SP) and *stratum radiatum* (SR) of the hippocampus (Figure 7A,B, respectively), there were no main genotype differences in the mean number and size of α4, α5, and α6 clusters (All *p* > 0.05). However, in both subregions of the hippocampus, the distribution of the size of α4-containing clusters showed a significant rightward shift in *mdx* mice compared with WT (SP: *p* < 0.0001; SR: *p* < 0.001), indicating a larger proportion of big clusters containing the α4 subunit in *mdx* mice. This was not observed for α5 and α6-containing clusters.

**Figure 8:**
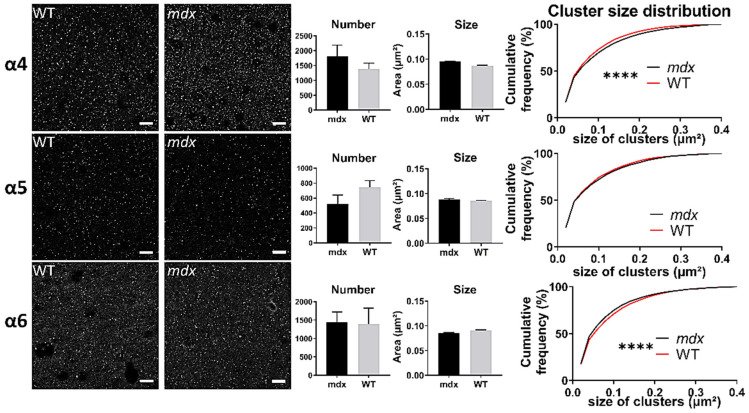
Immunohistological analysis of the α4–6 subunits contained in extrasynaptic GABA_A_Rs in the cerebellum. Analysis in the MCL of cerebellum. For each subunit and for both genotypes, from left to right: Representative sample confocal laser scanning microscope images, mean number of clusters, mean cluster size, and distribution curve of the cluster sizes expressed as cumulative frequency (%). Scale bar: 10 µm. **** *p* < 0.0001.

In the cerebellar molecular cell layer (MCL) (Figure 8), the mean number and mean size of clusters containing α4, α5, and α6 subunits were comparable in the two genotypes (*p* > 0.05). However, the distribution of cluster sizes in *mdx* mice showed a significant rightward shift for clusters containing the α4 subunit (*p* < 0.0001), and a leftward shift for those containing the α6 subunit (*p* < 0.0001). This suggests a larger proportion of big α4 subunit-containing clusters and of small α6-containing clusters.

**Figure 9:**
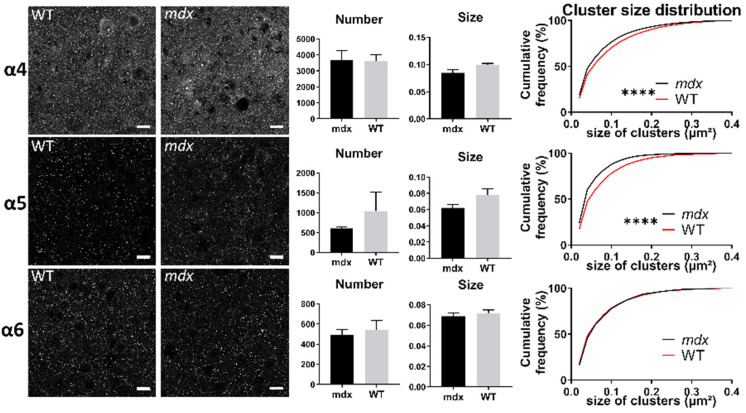
Immunohistological analysis of the α4–6 subunits contained in extrasynaptic GABA_A_Rs in the cortex. Analysis in the sensory-motor cortex. For each subunit and for both genotypes, from left to right: Representative sample confocal laser scanning microscope images, mean number of clusters, mean cluster size, and distribution curve of the cluster sizes expressed as cumulative frequency (%). Scale bar: 10 µm. **** *p* < 0.0001.

In the sensory-motor cortex (Figure 9), the mean number and size of clusters was comparable between genotypes (*p* > 0.05), but there was a significant leftward shift of the distribution of the sizes of clusters containing the α5 (*p* < 0.0001) and α6 (*p* < 0.005) subunits in *mdx* mice. This suggests that in this cortex, there was a larger proportion of small clusters containing the α5 and α6 subunits in *mdx* mice.

Table 2 summarizes the significant changes observed in the distribution of the size of clusters containing distinct GABA_A_R subunits.

## 3. Discussion

Many lines of evidence indicate that the absence of brain full-length dystrophin (Dp427) in DMD mouse models modifies the molecular machinery involved in the formation and long-term maintenance of central inhibitory connectivity. Dp427 loss particularly affects the number, size, and distribution of GABA_A_R clusters containing the α1 and/or α2 subunits within certain brain structures [5,39]. Preclinical studies have shown that Dp427 rescue by exon-skipping strategies in the adult brain of *mdx* mice has the potential to restore GABA_A_R clustering, as well as the associated excitatory-synapse plasticity and emotional disturbances [19,20,22,30,31]. This suggests that alterations of local inhibitory networks at least partly underlie the behavioral and cognitive deficits associated with DMD, and that changes in specific GABA_A_R subunits may serve as key readouts to evaluate the efficacy of genetic therapies. However, two studies demonstrated that extrasynaptic GABA_A_Rs may also be affected in *mdx* mice [23,29], implying a more complex pattern of alterations of multiple GABA_A_R subunits. In this study, we therefore used both in vivo and ex vivo approaches to further characterize the number of GABA_A_Rs and the expression of a variety of their subunits in several regions of the nervous system in *mdx* mice. One main objective was to characterize novel molecular outcome measures that could be used in preclinical studies to evaluate the efficacy of therapies targeting the CNS. Brain PET imaging using [^11^C]flumazenil showed that the density of GABA_A_Rs is unaffected by the absence of Dp427 in *mdx* mice, while the expression levels and/or distribution of both synaptic and extrasynaptic GABA_A_R subunits is differentially regulated in distinct brain structures and spinal cord.

### 3.1. Density of GABA_A_Rs

Our PET imaging study with [^11^C]flumazenil did not reveal any difference between *mdx* and WT mice regarding the total number of GABA_A_Rs in main brain structures that normally express Dp427, such as the hippocampus, cortex, and cerebellum. This seems in contrast with the results obtained in a single photon emitting computed tomography (SPECT) imaging study in DMD patients with a molecule with similar receptologic profile, [^123^I]iomazenil, which found a reduction in the density of GABA_A_Rs in the prefrontal cortex of these patients [40]. A difference between the human and mouse conditions cannot be ruled out. However, the lack of genotype identification in the human study did not allow for confirmation that the observed change was specifically due to the lack of Dp427, rather than being influenced by a cumulative deficiency of several dystrophins inducing additional reorganizations of synaptic networks [41,42]. Moreover, the focused analysis of the prefrontal cortex in the patient study does not provide information as to whether this could be generalized to other brain structures investigated in mice, and an apparent influence of aging on the measurements in patients suggests that additional adaptive factors were involved. Moreover, PET offers absolute quantification whereas SPECT only allows for semi-quantitative interpretation of brain images which may interfere with the interpretation of imaging data [43].

Our present results in *mdx* mice fully agree with previous studies showing that Dp427 loss does not affect the expression of the main GABA_A_Rs anchoring protein, gephyrin, which already suggested that the global density of GABA_A_Rs should not be affected in *mdx* mice. Even though extrasynaptic receptors may be clustered by additional mechanisms involving radixin [44], it was shown that [^11^C]-Flumazenil imaging reflects the density of all types of GABA_A_Rs, including both postsynaptic and extrasynaptic receptors [45,46]. This is a strong argument to conclude that Dp427 loss does not impact the total number of central GABA_A_Rs in mice. Our immunoblot study confirms and elaborates upon this finding, as all of the structures analyzed here show comparable expression of the γ2 subunit, a specific core component of synaptic GABA_A_Rs, and of the δ subunit that characterizes a majority of extrasynaptic receptors. We cannot exclude the possibility that a decrease in the number of receptors per synapse could be masked by a compensatory increase in synapse density, as we have previously shown that there are more inhibitory synapses in the dorsal CA1 hippocampal area of *mdx* mice [43]. In any case, the in vivo quantification of GABA_A_R density does not provide a translational tool to evaluate central GABAergic alterations in preclinical studies, because this parameter is not affected by Dp427 loss in *mdx* mice.

**Figure 10:**
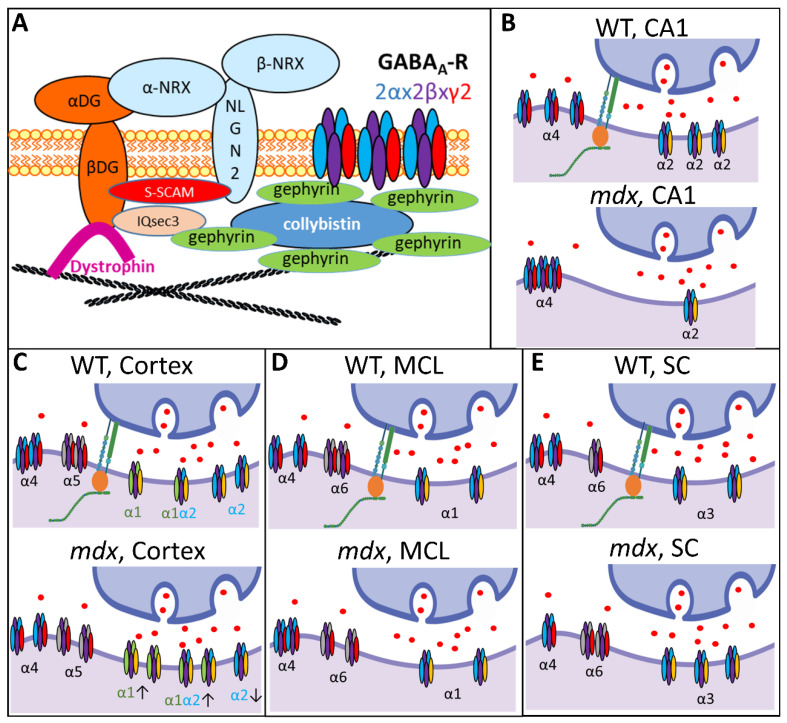
Brain dystrophin deficiency and the multiple alterations of GABA_A_R subtypes in distinct regions of the nervous system. (**A**) Diagram showing the interaction of the dystrophin-associated complex (α and β dystroglycans) with key postsynaptic scaffolding proteins (Neuroligin-2, neurexin, S-SCAM, IQsec3) involved in the regulation of GABA_A_R clustering in central inhibitory synapses. As indicated in top right corner, the different colors in GABA_A_Rs correspond to the α subunits (light blue), β subunits (purple) and γ2 subunits (red). (**B**–**E**) Schematic summary of the results obtained in this study comparing the expression and distribution of synaptic and extrasynaptic GABA_A_R subunits in WT and dystrophin-deficient *mdx* mice. The working models combine expression-level data from semi-quantitative immunoblots and distribution of cluster sizes from IF confocal analyses. The main type of α subunit contained in the displayed receptors is indicated (subunits’ colors are arbitrary, but the font color matches the color of the subunits in the drawing when their expression is changed in *mdx* mice). Arrows below isolated synaptic GABA_A_Rs in the *mdx*-cortex drawing indicate decreased (down) or increased (up) expression levels of specific α subunits. Adjacent receptors symbolize large clusters. CA1: Hippocampal CA1 area; MCL: molecular cell layer of cerebellum; SC: spinal cord.

As shown in Figure 10 (upper left panel), brain dystrophin in the PSDs of inhibitory synapses is associated with the dystroglycan complex, composed of extracellular α-dystroglycan (α-DG) that binds Neuroligin-2, and β-dystroglycan (β-DG) that binds the scaffolding protein S-SCAM and gephyrin-associated IQsec3 (or SynArfGEF) proteins. Dystrophin and dystroglycan appear to be dispensable for the gephyrin-dependent anchoring of GABA_A_Rs [4,6,47], which may explain why the total number of GABA_A_Rs is unaffected in *mdx* mice. However, Dp427 deficiency induces alterations of neuroligin-2 expression and might affect S-SCAM and IQsec3 as well, which may significantly modify the formation, maturation, and long-term maintenance of at least a subset of GABAergic synapses [5,39,48,49]. The reported decrease in the number of clusters containing the synaptic α1 and α2 subunits in *mdx* mice, as well as their higher sensitivity to selective activators of extrasynaptic receptors, could thus reflect complex changes in the proportion of specific GABA_A_R subtypes or modifications of the subunit assembly [18].

### 3.2. Expression of Synaptic GABA_A_Rs

Our precise semi-quantitative analysis of immunoblots demonstrated that the expression levels of synaptic subunits are differentially regulated in *mdx* mice, and also revealed that selective changes occur depending on the brain structure (Figure 10). Large variation in expression of the different GABA_A_R subunits may exist in the distinct CNS structures, which were analyzed in separate immunoblots to focus on the genotype differences. Putative changes in the relative expression of subunits between structures could not be estimated from these analyses and the genotype differences are therefore discussed for each structure separately.

In the hippocampus, we confirm a significant decrease of the α2 subunit expression, which was previously reported in two studies using quantitation of α2-containing cluster immunoreactivity in hippocampal sections [6,20]. This also partially correlates with another seminal study [50], which analyzed the expression of α1 and α2 subunits mRNAs in different brain structures of the *mdx* mouse and reported their decreased expression in the hippocampus. In the present study, we confirm a decrease in α2 expression in the hippocampus at the protein level, but we did not detect significant changes in the α1 subunit expression. Aside from putative methodological differences between the two studies, protein expression does not always strictly follow changes in transcript expression levels, due to multiple post-transcriptional regulations and putative systemic adaptations and remodeling of the GABAergic synaptic networks. We also report significant decreases in the expression of the β2 and β3 subunits. Because these two subunits can be associated with the α2 subunit, one may hypothesize a decrease in the number of synaptic receptors composed of α2β2γ2 and/or α2β3γ2 subunits, perhaps partly compensated by α3-containing receptors, as we observed a non-significant but noticeable increase (~50%) in the expression level of this subunit.

In the cerebellum, we found no difference in the expression of all subunits as a function of genotype. This is in line with previous studies showing that changes in α1 subunit expression in the cerebellum cannot be detected in immunoblots [24], confirming that the total number of receptors containing this subunit is not affected, while their ability to form large clusters is selectively impaired [6].

In the cortex, there was an overexpression of the α1 subunit and a downregulation of the α2 subunit, suggesting major changes in the main α1βγ and α2βγ synaptic GABA_A_Rs expressed at the inhibitory synapse. The opposing changes in expression of these two subunits suggested putative changes in the proportion of synapses expressing these receptor subtypes, or subtle modifications in the relative expression of these two GABA_A_R subtypes within the synapse. It is suspected that α1- and α2-GABA_A_Rs have distinct roles in the formation of GABAergic circuits in close interaction with the dystrophin-glycoprotein complex, but it was also shown that the absence of the α1 subunit in the hippocampus may induce a compensatory increase in α2-containing synapses and a reorganization of inhibitory circuits [16,48]. To further detail this observation, we performed a double immunohistochemical labeling of α1 and α2 and analyzed the putative colocalizations of clusters containing these subunits in the cortex. Interestingly, the two subunits colocalized in ~30% of GABA_A_R clusters in WT mice, and there was a significant increase by 10% of this colocalization in *mdx* mice. This suggests that the absence of dystrophin from inhibitory synapses is associated with an increased density of clusters that contain both α1βγ and α2βγ subtypes of GABA_A_Rs. Alternatively, these results may also fit with a working model in which changes in the expression of these subunits in *mdx* mice reflect the presence of hybrid receptors containing both α1 and α2, as suggested by others [18]. Several hybrid GABA_A_Rs containing heterologous α1/α2, α1/α3, and α2/α3 pairs of subunits have been characterized in the mammalian cortex and shown to include the synaptic γ2 subunit and a benzodiazepine-binding site [9,10,11]. It has been suggested that dystrophin may play a role in the stabilization of GABA_A_R clusters, perhaps by limiting their lateral diffusion along the postsynaptic membrane. To date, however, there is no known mechanism that directly involves dystrophin in the assembly of GABA_A_R subunits, and the present results might reflect an adaptation of inhibitory networks during synaptogenesis [16,48].

### 3.3. Expression of Extrasynaptic GABA_A_Rs

In our immunoblots analysis, the cervical spinal cord was the only structure showing significant changes in the expression level of extrasynaptic GABA_A_R subunits between genotypes. This was characterized by a decreased expression of α4 and an increased expression of α6, in addition to an increased expression of the synaptic α3 subunit. A non-specific effect of inflammation on peripheral inhibitory synapses cannot be ruled out. Indeed, in *mdx* mice muscle degeneration and regeneration has a retrograde inflammatory effect on the peripheral nervous system, including neurodegenerative processes that specifically reduce the synaptic activity of spinal alpha motoneurons [51]. This represents an overlooked feature of DMD pathophysiology, in which commutative mechanisms in the central and peripheral nervous systems may contribute to motor behavior deficits. However, we demonstrated here that Dp427 is expressed in the spinal cord, yet at a level 3–4 times lower than in brain structures. This suggests that these changes observed in *mdx* mice instead reflect a role played by dystrophin in the clustering of GABA_A_Rs in peripheral inhibitory synapses. Furthermore, the altered expression of extrasynaptic GABA_A_Rs in spinal cord might explain the enhanced behavioral sensitivity of *mdx* mice to gaboxadol, a pharmacological agent that binds preferentially to extrasynaptic subunits α4, α6, and δ [52]. We previously showed that *mdx* mice more rapidly display a loss of the righting reflex induced by gaboxadol, as do mice overexpressing α6 subunit-containing extrasynaptic receptors [23,53]. Because gaboxadol was administered intra-peritoneally in this previous study, a contribution of peripheral GABA_A_Rs was therefore possible. Importantly, we show that the α6 subunit expression is also increased in the spinal cord of *mdx* mice, which further suggests implication of peripheral α6-containing GABA_A_Rs in this phenotype [54].

Changes in extrasynaptic GABA_A_Rs in *mdx* mice cannot be limited to the spinal cord, as their higher sensitivity to gaboxadol was also demonstrated ex vivo by electrophysiological techniques in Purkinje neurons of acute cerebellar slices [29]. We therefore hypothesized that this phenotype is not associated with bulk changes in the expression level of extrasynaptic GABA_A_Rs, but rather with more discrete modifications of the size and/or distribution of specific extrasynaptic clusters. Using IF and confocal imaging of brain tissue sections, we detected significant changes between genotypes regarding the size of clusters containing the α4–6 extrasynaptic subunits in the hippocampus, cortex and cerebellum (Figure 10).

In the CA1 region of the hippocampus, the proportion of big clusters containing α4 was larger in *mdx* than in WT mice in both the neuronal and dendritic layers (SP and SR). In the sensory-motor cortex, however, *mdx* mice expressed a larger proportion of small clusters containing the α4 and α5 subunits. In the cerebellum, *mdx* mice were characterized by big α4 clusters and small α6 clusters. Interestingly, this shows that changes in the formation of extrasynaptic clusters are not restricted to the cerebellum in *mdx* mice, but may be differentially altered depending on the brain structure. Clustering alterations are not necessarily characterized by a reduced capacity to form large clusters, as larger clusters containing specific subunits were also detected in *mdx* mice, i.e., α4 in the hippocampus and cerebellum. This suggests that distinct structures underwent different reorganization of extrasynaptic GABA_A_Rs within local inhibitory circuits due to the absence of dystrophin. We may assume that such changes occur in extrasynaptic domains because these subunits normally concentrate extrasynaptically. However, future studies could consider other hypotheses, such as the possibility that the absence of Dp427 could favor specific mechanisms allowing extrasynaptic receptors to switch to synaptic domains. In addition to endocytic recycling and lateral diffusion, it has been proposed that a reservoir of extrasynaptic receptors might indeed play a role in the plasticity of inhibitory synapses by supplying the synaptic pool of GABA_A_Rs [55]. In any case, the changes observed here in *mdx* mice likely alter the physiological properties of extrasynaptic GABA_A_Rs and their sensitivity to specific pharmacological compounds such as gaboxadol [8,52]. This may also contribute to a variety of emotional, cognitive, and neuropsychiatric disorders [55,56,57,58,59,60,61] including those reported in *mdx* mice and associated with DMD, such as stress reactivity, anxiety, fear memory, and intellectual disability [23].

### 3.4. Concluding Remarks

We demonstrate that the absence of Dp427 brain dystrophin alters the expression level, distribution and subunit composition of multiple synaptic and extrasynaptic GABA_A_Rs in both central and peripheral inhibitory synapses. This strongly suggests that the role of dystrophin in GABAergic synapses is not limited to the stabilization and confinement of GABA_A_Rs in specific synaptic domains, but may also influence the combination of specific receptor subunits that determine their function and pharmacology.

The observed alterations in central synapses differ among brain structures, suggesting the establishment of variable adaptive processes during synaptogenesis and/or adult brain plasticity. Moreover, we cannot exclude that dystrophin may normally interact with only a subset of these subunits, and that part of the observed changes in *mdx* mice reflect compensatory adaptations within GABAergic network rather than a direct effect of dystrophin loss. Our results do not allow to discriminate cytosolic versus membrane-bound receptor subunits. Future studies comparing the total, membrane, and endocytic fractions might help to further determine whether dystrophin-dependent regulations take place at the plasma membrane or during earlier steps of subunits assembly. Nevertheless, the changes we report for extrasynaptic receptors, in line with previous studies showing a higher sensitivity to gaboxadol in *mdx* mice support the need to investigate putative alterations of the lateral diffusion of GABA_A_Rs. Analyses of their expression in membrane fractions would provide limited information regarding the lateral-diffusion hypothesis that should be addressed using specific technologies [62,63,64,65].

Our present study combining Western blotting and IF analyses of cluster distribution provides novel insights into the regulations of the GABAergic system in the dystrophin-deficient mouse, which encompass changes of both postsynaptic and extrasynaptic receptors. This should be further detailed in future studies, as it may open the way for innovative pharmacological interventions based on allosteric modulation of specific GABA_A_R subtypes. Importantly, we show that a relevant part of the molecular alterations reported here can be quantified using standard Western blots. This provides new outcome measurement tools that could be easily replicated in laboratories involved in preclinical mouse studies to evaluate the impact of molecular and pharmacological treatments on the central comorbidities associated with the DMD syndrome.

## 4. Materials and Methods

### 4.1. Animals

C57BL/10ScSn-*Dmd*^mdx^/J (*mdx*) dystrophin-deficient and C57BL/10ScSnJ littermate (WT) male mice were obtained by mating heterozygous *mdx* females with WT males. Siblings were kept in the same cage (two to five per cage) under a 12-h light-dark cycle (light on: 7:00 a.m.) with food and water ad libitum. Mouse genotype was verified by polymerase chain reaction. Both in vivo and ex vivo experiments were performed in adult male mice aged 3–5 months.

### 4.2. [^11^C]-Flumazenil PET Imaging: Acquisition Protocol and Imaging Data Analysis

PET imaging experiments were performed on an Inveon microPET scanner (spatial resolution ~1.6 mm; Siemens Healthcare, Knoxville, TN, USA) in anesthetized mice (1.5–2.5% inhaled isoflurane, weight 34.2 ± 3.7 g). [^11^C]flumazenil was formulated in 0.9% aqueous saline with 3% ethanol (*v*/*v*). The radiochemical purity of [^11^C]flumazenil was >98% and the molar activity at the time of injection was 6.8 ± 0.9 GBq.μmol^−1^. Data acquisition started immediately after intravenous bolus injection of [^11^C]flumazenil (6.8 ± 0.9 MBq) for 60 min.

Dynamic PET images were reconstructed using standard OSEM-2D reconstruction algorithm while correcting for radioactive decay, scatter, and attenuation. PET data analysis was performed using the PMOD software (version 3.8, PMOD Technologies Ltd., Zurich, Switzerland). Brain PET images were co-registered with an MRI template of the mouse brain to define selected regions of interest, consistent with the spatial resolution of the scanner [66]. Regional time-activity curves (TACs) were generated and expressed as percent of injected dose per volume (ID%/cm^3^). Specific binding of [^11^C]flumazenil to GABA_A_Rs in tested brain regions was estimated using the Logan-reference kinetic model. The regional binding-potential (BP_ND_) was estimated using the brain stem, a region with low expression of GABA_A_Rs, as a previously described reference region [34]. Parametric images describing [^11^C]flumazenil BP_ND_ were then generated for each animal. The parametric images obtained in each group (*n* = 5 WT and 6 *mdx* mice) were then compared using a statistical parametric mapping (SPM) and a voxel-to-voxel analysis (SPM8 software, London, UK). A brain mask was created from a [^11^C]flumazenil brain mouse template and applied to parametric maps to include cerebral voxels only. Statistical comparisons were then performed using an unpaired *t*-test design to detect significant difference in BP_ND_ between groups. A significance level threshold of 0.05 (uncorrected for multiple comparisons) and a minimum cluster size of 200 voxels were selected. Only the clusters that were significant at *p* < 0.05 levels (corrected for multiple comparisons) were considered.

### 4.3. Western Blot Analyses of GABA_A_R Subunits

Total protein extracts from *mdx* (*n* = 7–8) and WT littermate mice (*n* = 7) were obtained from dissected brain structures (cortex, hippocampus, cerebellum, and cervical spinal cord) treated with RIPA lysis and extraction buffer (ThermoFisher Scientific, Villebon Sur Yvette, France), complemented with SDS powder (5% final) (Bio-Rad, Marnes La Coquette, France). The total protein concentration was determined with the BCA Protein Assay Kit (ThermoFisher Scientific, Villebon Sur Yvette, France). Samples were denatured at 100 °C for 3 min and 25 μg of protein were loaded onto NuPAGE 4–12% Bolt Bis-Tris Plus Protein gels following manufacturer instructions (Invitrogen, Villebon Sur Yvette, France). Membranes were incubated with antibodies directed against various GABA_A_R subunits (Synaptic system, Gottingen, Germany; Merck, Molsheim, France) along with vinculin (Sigma Aldrich, St Quentin Fallavier, France) for normalization (Table 3). All samples of a given structure from both genotypes were deposited on the same gel in order to obtain a reliable quantification. Antibody signals were visualized using the Odyssey CLx system (Li-Cor, Homburg, Germany) and quantification was done using the Empiria Studio software (Li-Cor, Homburg, Germany). The specificity of the primary antibody was checked by omitting the primary antibody, which did not reveal unspecific bands at the expected molecular weight of the target proteins, and/or by using incubation with a control peptide to confirm the specific bands to be quantified (for the antibodies directed against the α1, α3, and α5 subunits). As described by the manufacturer (Synaptic system, Gottingen, Germany), the α2, β1, and β3 antibodies reveal several relatively close bands which are specific of the analyzed subunits, as previously validated using specific KO mice (α2) and/or control peptides (β1 and β3). Both bands were therefore quantified in this study. Data are presented as mean ± SEM. Statistical analyses were performed using the Mann–Whitney U test in the GraphPad 8 software (Prism, San Diego, CA, USA).

### 4.4. Semi-Quantitative Immunohistochemistry

Fresh-frozen brain cryosections of 30 µm thickness from 5 mice per genotype were collected onto Superfrost® Plus slides (Carl Roth, Karlsruhe, Germany), thawed for 2 min at room temperature (RT), fixed in acetone/methanol (1:1) for 5 min at −20 °C, washed in PBS, incubated first in a blocking solution for 45 min (10% normal goat serum, 0.3% Triton X-100, and 1% BSA), then overnight at 4 °C with the primary antibody (Dilutions: see Table 3), washed and incubated with the secondary antibody (Dilutions: see Table 3). Controls prepared by omitting the primary antibody showed no specific staining. In each genotype, five cryosections were used to analyze each GABA_A_R subunit (one section from *n* = 5 mice per genotype). All sections paired between *mdx* and WT mice were taken at equivalent stereotaxic coordinates, between Bregma −1.22 to −2.46 mm for coronal sections including hippocampus and cortex, and from lateral 0.12 to 1.20 mm for sagittal sections of the cerebellum [67]. Within each structure, images were taken at equivalent locations and exposure times using a laser scanning confocal microscope (Zeiss LSM 700, x63 objective; Zeiss, Rueil Malmaison, France). Typically, stacks of 8–9 images (1024 × 1024 pixels) spaced by 1 µm were recorded at a magnification of 99 nm/pixel. For quantification of GABA_A_R clusters, digital images were processed with the WCIF ImageJ imaging system (Rasband, W.S., ImageJ, U. S. National Institutes of Health, Bethesda, MD, USA) as follows [6,68,69]: The punctate IR for GABA_A_Rs representing presumptive postsynaptic clusters was analyzed semi-quantitatively in the SP and SR layers (10–100 µm below the SP) of the CA1 subfield of dorsal hippocampus, in layers II–VI of the sensory-motor cortex (same sections as for hippocampus) and in the MCL of the cerebellum (lobules 4–6, 10–150 µm from Purkinje-cell layer), which are known to be strongly immunopositive for dystrophin [6,38]. Quantification of GABA_A_R clusters was performed from a single confocal image taken in the middle of the stack. The minimal cluster size was arbitrarily set to 0.02 μm^2^, corresponding to two adjacent pixels at the magnification used. A threshold segmentation algorithm was used for automatic detection of clusters. The mean number and size of the clusters, as well as the distribution of cluster sizes, were analyzed within a total tissue surface of 10,000 μm^2^ per animal in each brain structure or subregion. Clusters double-labeled for the α1 and α2 GABA_A_R subunits were identified using all images of the stacks with the 3D colocalization analysis tool of the Imaris image analysis software (version 9, Bitplane, Zurich, Switzerland). Data presented as means ± SEM were statistically analyzed using the Mann–Whitney U test, while the distributions of the cluster sizes were compared using the Kolmogorov–Smirnov test with a significance threshold set at *p* < 0.005 (GraphPad 8 software, Prism software, San Diego, CA, USA).

### 4.5. Detection of Dystrophin in Spinal Cord Using the Automated Jess Western Blot System

Detection of dystrophin expression in the spinal cord was performed using the capillary Western immunoassay Jess system based on the protocol previously described by Beekman et al. [70] and according to the manufacturer’s instructions (Bio-Techne, Minneapolis, MN, USA), using a 66 ± 440 kDa Separation Module (ProteinSimple SM-W008), the Anti-Rabbit Detection Module (ProteinSimple DM-001), and the Anti-Mouse Detection Module (ProteinSimple DM-002). Protein samples (1 µg) were prepared in sample buffer from the Separation Module, then mixed with Fluorescent Master Mix and heated at 95 °C for 5 min. The samples, blocking reagent (antibody diluent), primary antibodies (in antibody diluent), HRP-conjugated secondary antibodies, and chemiluminescent substrate were pipetted into the plate (part of Separation Module). Dystrophin expression was detected with a combination of primary antibodies including the mouse monoclonal antibodies, NCL-DYS1 (1/25, Leica Biosystems, Nanterre, France), MANDYS1(3B7) (1/25, Developmental Studies Hybridoma Bank—DSHB, Iowa City, IA, USA) and MANEX58D(7G2) (1/25, Developmental Studies Hybridoma Bank—DSHB), and the rabbit monoclonal ab154168 (1/1000, Abcam, Paris, France). Vinculin was detected with the mouse monoclonal anti-vinculin hVin-1 antibody (1/100, Sigma) as normalization control. Instrument default settings were as follows: stacking and separation at 475 V for 30 min; blocking reagent for 5 min, primary and secondary antibodies for 30 min each; luminol/peroxide chemiluminescence detection for ~15 min. The resulting electropherograms were inspected to check whether automatic peak detection required any manual correction. A 6-point calibration curve was loaded, made of a mix of WT and dystrophin-deficient control lysates to obtain defined percentages of dystrophin (0, 5, 10, 15% or 0, 10, 15, 30% of corresponding WT tissues) as previously described for our Western blot analyses [30].

## Figures and Tables

**Table 1:** Summary of Western blot data. Main increases (upward arrows) and decreases (downward arrows) in the expression level of the different GABA_A_R subunits in *mdx* mice are shown for hippocampus (HIP), cerebellum (CBL), cortex (CX), and spinal cord (SC). Probabilities are shown for non-significant (marginal) differences and significant genotype differences are indicated by asterisks (* *p* < 0.05, ** *p* < 0.01).

	Western Blot Quantification			
	HIP	CBL	CX	SC
α1	-	-	** ↑	-
α2	* ↓	-	* ↓	-
α3	-	-	-	* ↑
α4	-	-	-	* ↓
α5	-	-	-	-
α6	-	-	-	* ↑
β1	-	-	0.07↓	-
β2	** ↓	-	-	-
β3	** ↓	-	-	-
γ2	-	-	0.07↑	-
δ	-	-	0.05↓	-

**Table 2:** Summary of immunohistochemical detection of subunits contained in presumed extrasynaptic GABA_A_Rs. No genotype differences were found for the mean number (Number) and mean size (Size) of clusters containing α4, α5, and α6 subunits. Main shifts in the distribution curves of the cluster sizes are shown. Upward arrows indicate a rightward shift reflecting a larger proportion of big clusters; downward arrows indicate a leftward shift reflecting a larger proportion of small clusters. **** *p* < 0.0001, *** *p* = 0.0007.

**HIP (SP)**	**Number**	**Size**	**Distribution**	**HIP (SR)**	**Number**	**Size**	**Distribution**
α4	-	-	**** ↑	α4	-	-	*** ↑
α5	-	-	-	α5	-	-	-
α6	-	-	-	α6	-	-	-
**CBL**	**Number**	**Size**	**Distribution**	**CX**	**Number**	**Size**	**Distribution**
α4	-	-	**** ↑	α4	-	-	**** ↓
α5	-	-	-	α5	-	-	**** ↓
α6	-	-	**** ↓	α6	-	-	-

**Table 3:** List of antibodies used in Western blots (WB) and immunohistochemistry (IHC).

**Primary Antibodies**					
**Target**	**Species**	**Dilution WB**	**Dilution IHC**	**Company, Cat.No**	
Vinculin	Mouse	1/50	-	Sigma Aldrich, V9131	
α1 subunit	Guinea pig	1/500	1/500	Synaptic System, 224 204	
α2 subunit	Rabbit	1/1000	1/1000	Synaptic System, 224 102	
α3 subunit	Rabbit	1/500	-	Synaptic System, 224 303	
α4 subunit	Rabbit	1/100	1/100	Synaptic System, 224 402	
α5 subunit	Rabbit	1/500	1/500	Synaptic System, 224 502	
α6 subunit	Rabbit	1/500	1/500	Synaptic System, 224 603	
β1 subunit	Guinea pig	1/500	-	Synaptic System, 224 705	
β2 subunit	Guinea pig	1/500	-	Synaptic System, 224 805	
β3 subunit	Mouse	1/500	-	Synaptic System, 224 411	
γ2 subunit	Rabbit	1/1000	-	Synaptic System, 224 003	
δ subunit	Mouse	1/1000	-	Merck, 05-474	
**Secondary Antibodies**					
**Target**	**Species**	**Use**	**Dilution**	**Fluorophore**	**Company, Cat.No**
Mouse	Goat	WB	1/2000	IRDye 800CW	Li-Cor Biosciences, 926-32210
Rabbit	Goat	WB	1/2000	IRDye 800CW	Li-Cor Biosciences, 926-32211
Guinea Pig	Donkey	WB	1/2000	IRDye 800CW	Li-Cor Biosciences, 926-32411
Rabbit	Goat	IHC	1/400	Alexa 488	Invitrogen, A-11070
Guinea Pig	Goat	IHC	1/400	Alexa 555	Invitrogen, A-21435

## Data Availability

The data used to support our findings are included within the article and as supplementary data.

## Supplementary Materials

The supporting information can be downloaded at: https://www.mdpi.com/article/10.3390/ijms232012617/s1.
