# Supplementary material for: Abnormal Expression of Synaptic and Extrasynaptic GABAA Receptor Subunits in the Dystrophin-Deficient mdx Mouse
Source: Int J Mol Sci. 2022 Oct 20;23(20):12617. doi: 10.3390/ijms232012617 (PMC9604073; doi:10.3390/ijms232012617)
Supplement: Supplementary file 1 [file ijms-23-12617-s001.zip › ijms-1845994-supplementary.pdf]

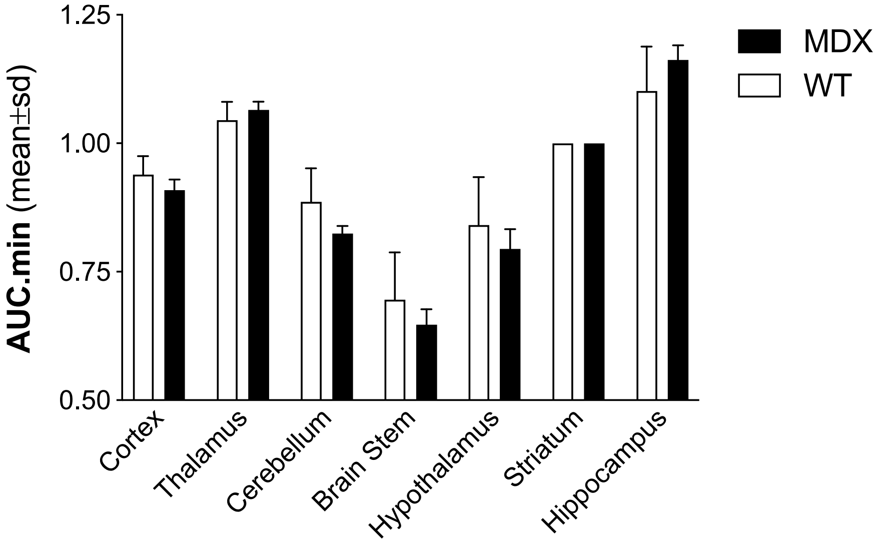

Supplementary figure S1. In vivo [11C]flumazenil PET brain imaging. Binding potential (BP_{ND}) to $GABA_A$ receptors in cortex, thalamus, cerebellum, brain stem, hypothalamus, striatum and hippocampus, estimated using the striatum as a reference region. Data are shown as mean ± S.D. Difference in BP_{ND} between wild-type and *mdx* mice was not significant ($p>0.05$).

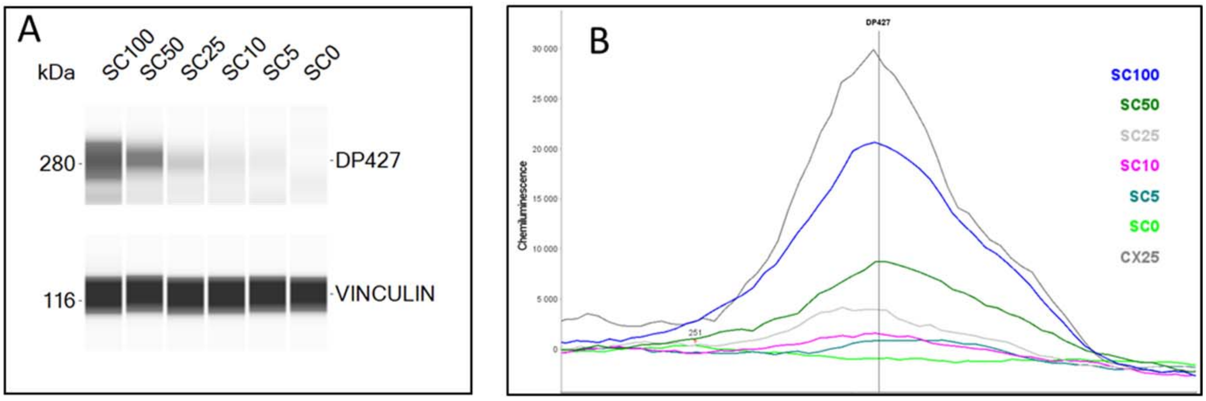

Supplementary figure S2. Detection of Dp427 in spinal cord. Virtual blot (A) and electropherogram (B) representing the Dp427 signal obtained in lysates from spinal cord containing 100% (SC100, blue), 50% (SC50, dark green), 25% (SC25, light grey), 10% (SC10, pink), 5% (SC5, turquoise) or 0% (SC0, light green) of WT dystrophin. In B, expression levels are compared to the levels obtained in a cortex sample containing 25% of wt dystrophin (Cx25, dark grey curve).
